# STN-DBS Reduces Saccadic Hypometria but Not Visuospatial Bias in Parkinson's Disease Patients

**DOI:** 10.3389/fnbeh.2016.00085

**Published:** 2016-05-03

**Authors:** Petra Fischer, José P. Ossandón, Johannes Keyser, Alessandro Gulberti, Niklas Wilming, Wolfgang Hamel, Johannes Köppen, Carsten Buhmann, Manfred Westphal, Christian Gerloff, Christian K. E. Moll, Andreas K. Engel, Peter König

**Affiliations:** ^1^Institute of Cognitive Science, University of OsnabrückOsnabrück, Germany; ^2^Medical Research Council Brain Network Dynamics Unit, University of OxfordOxford, UK; ^3^Nuffield Department of Clinical Neurosciences, John Radcliffe Hospital, University of OxfordOxford, UK; ^4^Department of Neurophysiology and Pathophysiology, University Medical Center Hamburg-EppendorfHamburg, Germany; ^5^Department of Neurosurgery, University Medical Center Hamburg-EppendorfHamburg, Germany; ^6^Department of Neurology, University Medical Center Hamburg-EppendorfHamburg, Germany

**Keywords:** deep brain stimulation, subthalamic nucleus, visual attention, neglect, oculomotor control, unilateral stimulation, basal ganglia, viewing bias

## Abstract

In contrast to its well-established role in alleviating skeleto-motor symptoms in Parkinson's disease, little is known about the impact of deep brain stimulation (DBS) of the subthalamic nucleus (STN) on oculomotor control and attention. Eye-tracking data of 17 patients with left-hemibody symptom onset was compared with 17 age-matched control subjects. Free-viewing of natural images was assessed without stimulation as baseline and during bilateral DBS. To examine the involvement of ventral STN territories in oculomotion and spatial attention, we employed unilateral stimulation via the left and right ventralmost contacts respectively. When DBS was off, patients showed shorter saccades and a rightward viewing bias compared with controls. Bilateral stimulation in therapeutic settings improved saccadic hypometria but not the visuospatial bias. At a group level, unilateral ventral stimulation yielded no consistent effects. However, the evaluation of electrode position within normalized MNI coordinate space revealed that the extent of early exploration bias correlated with the precise stimulation site within the left subthalamic area. These results suggest that oculomotor impairments “but not higher-level exploration patterns” are effectively ameliorable by DBS in therapeutic settings. Our findings highlight the relevance of the STN topography in selecting contacts for chronic stimulation especially upon appearance of visuospatial attention deficits.

## Introduction

Patients with Parkinson's disease develop a wide range of impairments other than the well-known skeletomotor symptoms. Among them, abnormal eye-movements and deficient spatial attention can potentially impair the sampling of visual information and impact patients' quality of life.

Previous research has shown that ocular movements are affected in Parkinson's disease (reviewed in Pinkhardt and Kassubek, 2011; Stuart et al., 2014). The most common finding is a reduction of saccade amplitudes and an increase in the latency to initiate a saccade. These changes are more evident for tasks with a stronger volitional component, like memory or antisaccade procedures, than for tasks of a more reflexive type. In how far these findings are related to impairment of general attentional and oculomotor control is still not well understood.

Overt and covert visuospatial biases have mainly been reported for patients with left hemibody onset of motor symptoms (Villardita et al., 1983; Starkstein et al., 1987; Ebersbach et al., 1996; Lee et al., 2001; Laudate et al., 2013). Left-dominant expression of parkinsonian symptoms is related to more severe neurodegeneration in the contralateral (right) basal ganglia and is often accompanied by orientation biases toward the right. In comparison, these attentional biases of Parkinson's disease patients are less pronounced than in the case of neglect syndrome (Halligan et al., 1990; Lee et al., 2001). However, even subtle attentional deficits may exert significant impact on daily life activities, and may partly explain behavioral anomalies, such as poor driving performance (Uc et al., 2006; Classen et al., 2014) or predisposition to bump into objects or doorways (Davidsdottir et al., 2005). Therefore, the study of visuo-spatial deficits is of high clinical relevance. Additionally, it provides an opportunity to improve our understanding of how the basal ganglia are involved in eye-movement and attentional control.

The effects of bilateral deep brain stimulation (DBS) of the subthalamic nucleus (STN) on oculomotor parameters, like saccade amplitude and latency, has been tested with tasks allowing only very specific patterns of movement. Reflexive tasks involving saccades to targets appearing in predictable positions suggest that STN-DBS improves saccade latencies and amplitudes (Sauleau et al., 2008; Temel et al., 2008, 2009; Fawcett et al., 2010; Yugeta et al., 2010; Antoniades et al., 2012a,b). However, such effects have not been found consistently (Rivaud-Péchoux et al., 2000; Lohnes and Earhart, 2012). STN-DBS can improve amplitude and latency of saccades also in memory and anti-saccade tasks (Rivaud-Péchoux et al., 2000; Fawcett et al., 2010; Yugeta et al., 2010), relying again on simple stimuli and specific movement goals. It is less clear whether similar improvements can be observed in settings close to real world situations when patients are allowed to explore freely, and whether stimulation affects attentional deficits.

The effects of STN-DBS on attentional deficits have previously been tested in a reaction time task (Witt et al., 2006). A recent study also examined exploration behavior during free-viewing of complex scenes (Schmalbach et al., 2014). However, these studies focused on unilateral and bilateral stimulation conditions but did not include comparisons to a control group. The results showed that only exclusive stimulation of the left electrode resulted in a small attentional and viewing bias to the right hemifield. Surprisingly, in the baseline condition, where the stimulation was switched off, patients showed no attentional bias in the reaction time task. Unilateral left stimulation therefore introduced a bias not present without stimulation. In the free-viewing task, no baseline condition (without DBS) was recorded, in which biases might have been easier to detect because of decreased compensatory cognitive control in comparison to the reaction time task. On the contrary, it has been reported that oculomotor deficits were less obvious during the exploration of line-drawings of increasing complexity (Matsumoto et al., 2011). Thus, unconstrained exploration of complex stimuli might generally result in compensation of motor and attentional deficits in Parkinson's disease. This raises the question whether patients with predominantly left-sided symptoms are biased during exploration of complex visual stimuli at all—a task closer to the demands of oculomotor control patients are facing in daily activities.

In the present study, we evaluated oculomotor performance and aspects of visual attention in Parkinson's disease patients with left symptom onset during a free-viewing task. Deficits in attention could affect the auditory or sensory domain as well, however, our study specifically evaluates deficits apparent during visual exploration. In order to establish visuospatial deficits inherent to the disease, we measured patients during a baseline condition (without DBS) and compared them to a group of age-matched control subjects. Additionally, we evaluated bilateral and unilateral STN-DBS. Similar to non-human primates (Matsumura et al., 1992), the oculomotor territory of the human STN is located within its ventral region (Fawcett et al., 2005). In contrast to previous studies, in which unilateral DBS was directed at dorsal skeletomotor STN territories (Witt et al., 2006; Schmalbach et al., 2014), we attempted to selectively manipulate activity within the ventral oculomotor STN region and the subjacent substantia nigra pars reticulata (SNr).

We evaluated three hypotheses under these conditions. First, we hypothesized that oculomotor and attentional deficits observed in simple well-defined tasks should also be present during free-viewing of complex scenes. Specifically, patients without DBS are expected to make shorter saccades with a viewing bias to the right in comparison with controls. Second, we hypothesized that bilateral clinical stimulation, in addition to the known and desired clinical effects, would improve these deficits. And third, we predicted that unilateral stimulation in the ventral STN region would bias exploration toward the contralateral site.

## Materials and methods

### Participants

We collected eye movement data of 19 Parkinson's disease patients with left hemi-body symptom onset, and of 19 age-matched healthy control subjects. Two patients and two controls were excluded because the two patients participated only in one of our four experimental conditions, and the control subjects' calibration accuracy was insufficient. Three of the 17 remaining patients participated only in two of four conditions: during therapeutic stimulation and when stimulation was OFF. Handedness was evaluated with the Edinburgh handedness inventory (Oldfield, 1971). All participants were right-handed except for one ambidextrous patient. Demographic and clinical data are presented as mean ± SD in this section and in Table 1. Mean age was 56.8 ± 10.9 years for patients (12 male) and 56.8 ± 11.6 years for control subjects (9 male). Two patients and two control subjects showed deficient color vision according to the Ishihara Color Vision test. Patients were generally suffering from advanced Parkinson's disease (mean time post-diagnosis = 12.2 ± 6.4 years; mean Levodopa equivalent daily dose = 867 ± 284 mg, see Tomlinson et al., 2010; median UPDRS-III in DBS OFF = 34 ± 21 IQR) and had undergone bilateral stereotactic implantation of DBS electrodes in the STN (model DBS 3389, Medtronic Inc., Minneapolis, MN, USA) on average 2.4 ± 1.9 (range 0.3–7.5) years before the experimental investigations. Before surgery (≤ 7 days) all patients showed a significant improvement of the UPDRS-III score following intake of levodopa (median improvement = 63%; median motor score OFF = 39.5 ± 20 IQR vs. ON = 14.5 ± 9 IQR; Wilcoxon signed rank test, *p* < 0.001). For three patients the pre-operative UPDRS scores were not available. For the purpose of the pre-operative levodopa challenge, dopamine agonists and dopamine degrading inhibitors treatment was stopped > 7 days before the operation and substituted with an equivalent levodopa dose. The degree of symptom lateralization is quantified by the fraction of left-sided symptoms relative to symptoms on both sides, which was on average 64.8% (Table 1). Values around 50% show that as a result of disease progression symptoms have appeared bilaterally and become more symmetric.

**Table 1:** **Demographic information and disease history of patients (left) and age-matched control subjects (two rightmost columns)**.

**ID**	**Age**	**Gender**	**LED**	**PD Dur**	**DBS Dur**	**Pre-op UPDRS**		**Post-op UPDRS**			**Asymmetry**	**Therap. Stim Right**	**Therap. Stim Left**	**veR Volt**	**veL Volt**	**CO Age**	**Gender**
						**Dopa OFF**	**Dopa ON**	**DBS OFF**	**DBS ON**	**Rec. in Dopa OFF**							
1	32	M	631	2.0	0.8	44.0	25.0	13.0	6.0	No cond	73.3	−10/+11, 60, 130, 2.5 V	−2/+C, 60, 130, 2.3 V	2.8	1.5	30	m
2	42	M	567	7.5	2.1	40.0	24.0	47.0	21.5	No cond	54.9	−9/−10/+C, 60, 180, 2.0 V	−1/−2/+C, 60, 180, 2.1 V	N/A	N/A	40	m
3	45	M	1249	7.5	0.3	N/A	N/A	39.5	10.0	No cond	61.4	−10/+C, 60, 130, 2.7 V	−2/+C, 60, 130, 2.3 V	2.6	4.0	51	f
4	48	F	1082	12.0	0.8	34.0	19.0	34.0	14.0	No cond	66.7	−9/+C, 60, 130, 3.8 V	−2/+C, 60, 130, 2.8 V	2.4	2.0	51	m
5	49	M	809	9.0	2.3	26.0	7.0	32.0	7.5	veR, veL	77.8	−10/+C, 60, 90, 3.2 V	−2/+C, 60, 90, 2.0 V	2.4	5.0	53	f
6	53	M	624	0.0	0.3	50.0	17.0	48.0	35.0	veR, veL	53.1	−10/+C, 60, 130, 2.7 V	−2/+C, 60, 130, 2.7 V	1.4	2.8	53	m
7	58	F	1341	12.0	2.0	67.0	10.0	14.5	14.5	No cond	93.8	−10/+C, 60, 150, 2.6 V	−2/+C, 60, 150, 2.5 V	0.8	1.6	54	m
8	58	M	1203	20.5	3.0	54.0	10.0	22.5	18.5	No cond	45.2	−9/−10/+C, 60, 130, 3.0 V	−1/−2/+C, 60, 130, 3.3 V	3.4	4.3	54	f
9	60	M	1027	19.8	3.8	57.0	17.0	45.5	24.5	All cond	60.9	−8/+C, 60, 160, 2.0 V	−1/+C, 60, 160, 2.4 V	2.5	2.9	56	f
10	60	F	967	22.5	5.8	N/A	N/A	46.5	46.5	All cond	47.2	−10/+C, 90, 130, 3.9 V	−1/+C, 60, 130, 3.6 V	N/A	N/A	56	m
11	60	F	1038	8.5	1.8	39.0	6.0	42.0	42.0	No cond	65.4	−10/+9, 90, 130, 3.2 V	−2/+C, 60, 130, 2.0 V	2.8	1.4	57	f
12	60	M	550	10.0	3.2	N/A	N/A	55.0	27.0	veR, veL	55.3	−9/+C, 60, 180, 4.0 V	−1/+C, 60, 180, 4.5 V	3.2	2.4	61	m
13	62	M	926	11.5	1.3	51.0	44.0	52.0	23.0	No cond	53.9	−10/+C, 60, 160, 3.7 V	−2/+C, 60, 160, 3.5 V	1.6	3.2	63	m
14	65	M	566	16.7	3.0	15.0	9.0	25.0	9.5	No cond	75.6	−10/−9/+C, 60, 130, 3.9 V	−1/+C, 60, 130, 1.5 V	3.2	3.2	68	f
15	69	F	508	15.2	0.8	31.0	17.0	26.0	23.5	No cond	89.5	−10/+9, 60, 180, 2.5 V	−2/+C, 60, 180, 2.8 V	N/A	N/A	68	f
16	72	m	1111	12.2	2.0	29.0	11.0	27.5	15.5	No cond	64.3	−10/+9, 60, 130, 4.0 V	−2/+1, 60, 130, 3.5 V	3.6	4.0	76	f
17	73	m	537	20.5	7.5	31.0	12.0	31.0	24.0	No cond	63.0	−10/+C, 60, 130, 3.1 V	−2/+C, 60, 130, 3.1 V	4.0	5.6	76	m

LED = Levodopa equivalent daily dose (conversion factors after Tomlinson et al. (2010); PD Dur = Disease duration in years past diagnosis; DBS Dur = DBS Duration (years past electrode implantation); UPDRS = UPDRS-III score; Dopa = Dopaminergic medication; Rec. in Dopa OFF = Conditions recorded in Dopa OFF; Asymmetry = Symptom severity of the left side relative to severity on both sides (UPDRS-III 20−26 left-sided items/[left-sided items + right-sided items] ^*^ 100), Therap. Stim Right/Left = Therapeutic stimulation settings of the right or left electrode (Negative poles/positive pole [C = case, 0 and 8 are the ventralmost contacts for the left and right electrode respectively], pulse width (μs), frequency (Hz), intensity (V)), veR Volt = Voltage during unilateral ventral right stimulation; veL Volt = Voltage during unilateral ventral left stimulation; CO = Control subjects.

We obtained the present data in two different locations using the same equipment. All healthy control subjects and 13 patients were recorded at the Dept. of Neurophysiology and Pathophysiology at the University Medical Center Hamburg-Eppendorf, Germany. Four additional patients were recorded at a neurologist's practice (Dr. med. Oehlwein). All participants gave their written informed consent to participate in this study and were paid 10€ per hour for their participation. Our study complied with Helsinki Declaration guidelines and was approved by the local ethics committee (Nr. PV4298, Ethik-Kommission der Ärztekammer Hamburg).

### Experimental design

Participant's eye movements were recorded monocularly using a remote video-oculographic eye tracker system (EyeLink 1000, 500 Hz sampling rate, SR Research Ltd., Mississauga, Canada). Subjects were seated centrally in front of a 24″ flatscreen monitor at an eye-screen distance of 65 cm. Patients' average calibration error was 0.60° ± 0.38° (controls, 0.48° ± 0.19°).

Patients performed the visual exploration task described below in four different DBS conditions. For the baseline condition, stimulation was switched off (OFF). The bilateral condition employed standard therapeutic stimulation parameters (ON), and the remaining two experimental conditions consisted of unilateral monopolar stimulation of the most ventral DBS electrode contacts (unilateral ventral left, veL; unilateral ventral right, veR).

For all conditions in which stimulation was active, the pulse width and stimulation frequency remained unchanged from patients' clinical settings. In the unilateral ventral stimulation conditions, the voltage was adjusted as follows: First, the threshold for the occurrence of persisting side effects was determined clinically (e.g., stimulation-induced paresthesias or tetanic muscle contractions). Table 2 provides a summary of the side effects encountered. Stimulation voltage was then reduced by 20% of the previously determined side-effect threshold. Due to this procedure neither patients nor experimenters were blinded to the conditions. Stimulation intensities did not differ significantly between the two unilateral ventral stimulation conditions [veL: 3.14 ± 1.31 V; veR: 2.62 ± 0.88 V, paired *t*-test: *t*_(13)_ = 1.62, *p* = 0.13]. Conditions were randomized such that the orders of ON and OFF, and the order of veL and veR were balanced across patients. For eight patients the very first recording was in ON due to time constraints, whereas the other nine patients were measured first either in OFF (3 patients), veR (3 patients) or veL (3 patients). Recordings started at least 30 min after DBS parameters were changed.

**Table 2:** **Electrode positions (mm)**.

**ID**	**LEFT**							**RIGHT**						
	***x***	***y***	***z***	***SE***	**%Ri**	**InitB**	**ΔSac**	***x***	***y***	***z***	***SE***	**%Ri**	**InitB**	**ΔSac**
1	−11.9	−13.4	−7.1	TC, DYS	36.8	−1.01	1.1	10.4	−14.0	−7.1	PAR, DYS	44.3	−0.08	0.0
3	−11.9	−14.9	−6.9	TMC, DA	85.3	0.62	0.0	11.8	−14.6	−7.2	TMC	26.9	−1.32	0.2
4	−10.0	−13.5	−9.5	DYS, TC, MO	51.9	−1.19	0.2	10.3	−13.6	−6.4	PAR, DA	56.8	−0.52	0.2
5	−9.4	−14.4	−7.3	PAR	71.0	2.42	−0.3	10.6	−15.8	−7.1	PAR	58.6	1.97	−0.5
6	−11.1	−14.1	−6.9	PAR	47.5	0.14	0.2	11.7	−12.2	−8.1	PAR	60.6	0.88	−0.2
7	−8.4	−14.2	−11.0	PAR, DA	59.6	2.97	−0.6	11.0	−13.6	−6.7	DA	56.2	3.91	−0.2
8	−11.9	−12.2	−8.1	DYS, AUT	54.3	−0.50	−0.2	12.1	−14.0	−7.6	PAR, AUT	51.0	1.32	−0.1
9	−10.8	−13.8	−11.3	N/A	49.6	−1.25	0.8	9.4	−16.6	−8.4	N/A	50.0	−1.24	1.1
11	−10.2	−15.3	−8.4	TC, PAR, DYS	42.7	−1.61	0.3	12.7	−13.3	−7.8	MO, TMC	40.3	−2.96	−0.7
12	−6.0	−15.6	−12.6	PAR	80.4	3.60	0.5	11.0	−10.6	−5.9	PAR	76.7	2.36	0.2
13	−10.9	−12.8	−6.7	TMC, DYS, PAR, DA	40.7	−2.95	1.0	10.6	−14.5	−6.4	TC, DA, PAR	85.0	1.34	3.4
14	−9.0	−16.3	−7.7	PAR, DA	79.6	5.64	0.5	11.9	−14.6	−5.2	PAR, DA	69.0	1.60	1.1
16	−8.5	−15.6	−6.1	AUT, PAR	73.0	1.14	0.2	9.8	−15.1	−4.6	AUT, PAR	40.6	0.21	−0.5
17	N/A	N/A	N/A	N/A	57.1	−0.64	−0.8	N/A	N/A	N/A	N/A	55.1	−0.42	−1.1

*x = medio-lateral axis (+: right, −: left), y = antero-posterior axis, z = dorso-ventral axis. %Ri = % right side explored, InitB = initial bias (° visual angle, negative values are left of the midpoint), ΔSac = saccade length difference (right − left direction). Side effect (SE) abbreviations: DA = dysarthria; PAR = paresthesia; DYS = dyskinesia; TC = torticollis; TMC = tetanic muscle contractions; AUT = autonomic side effects (dizziness or lightheadedness); MO = mood change; N/A = not available. Patients were ordered by age starting with the youngest as in Table 1. Electrode positions of patient 17 could not be reconstructed because the quality of the scan was insufficient*.

**Figure 1:**
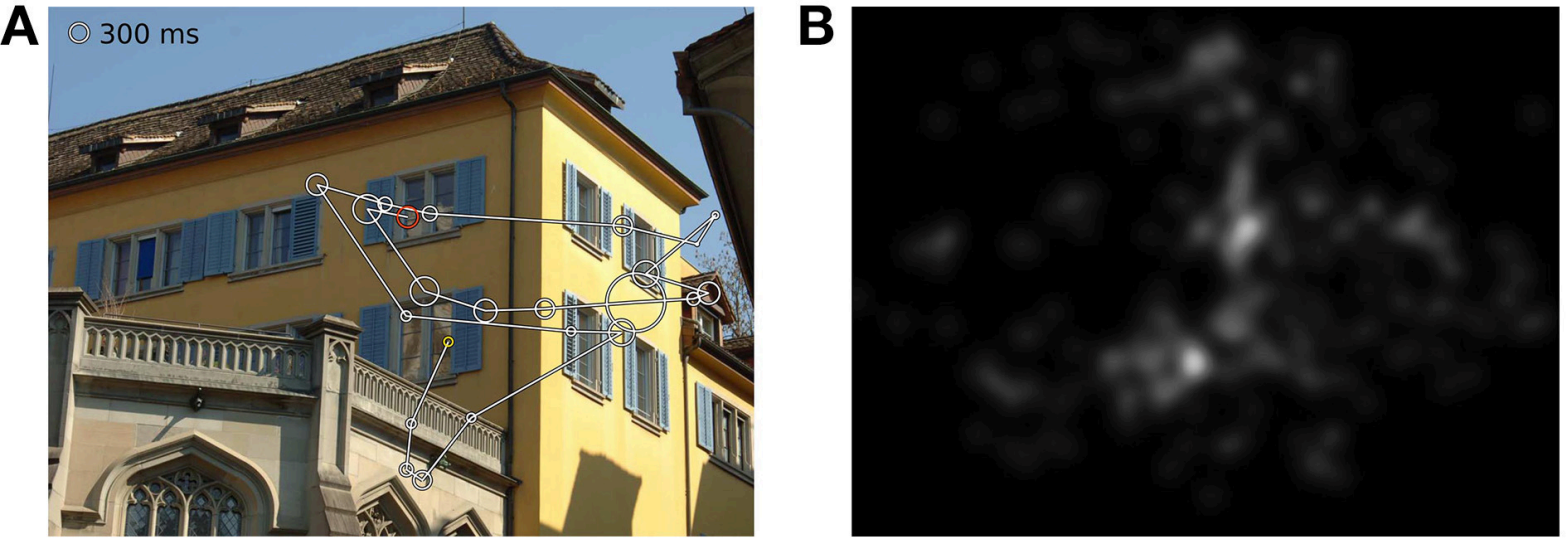
**(A)** Scanpath of one subject in one trial, **(B)** fixation density map of all patients viewing this image. The yellow circle depicts the first fixation and the red circle the last one. Circle sizes illustrate fixation durations.

In the first part of the experiment, participants carried out a visual search task (10–20 min) that we will report on separately. This was followed, after a short break, by the free-viewing experiment presented here. Participants were instructed to examine pictures as if visiting a museum or viewing a picture book. Completion of the free-viewing task took 6–7 min excluding calibration. In every stimulation condition, 35 images of natural (e.g., flowers, forests) or urban scenes (e.g., streets, houses) were displayed for 8 s each. Each stimulus onset was triggered by the experimenter to ensure that patients were fixating a dot presented in the middle of the screen prior to each trial. An example image with the scan-path from one subject and a fixation density map depicting the pooled patient data are shown in Figures 1A,B. In order to avoid viewing biases caused by an asymmetrical distribution of image content, we randomly presented images from a pre-selected pool of pictures. The images were balanced with respect to averaged spatial fixation behavior observed in previous experiments with healthy subjects (Ossandón et al., 2014).

Ventral subthalamic DBS can result in restricted eye motility due to accidental stimulation of the oculomotor nerve (Bejjani et al., 2002). Therefore, we carefully assessed eye motility for each experimental condition and asked the patients if they experienced double vision. Clinical examination of eye movements was routinely carried out before the start of each session. Furthermore, we took photographs after completion of each experimental condition while participants shifted their gaze into nine different directions without moving their head (see Supplementary Figure 1). Importantly, none of our patients reported double vision or showed restricted eye motility.

Finally, at the end of each condition block, an experienced movement disorder specialist (CKM) evaluated patients' motor impairments according to the motor subsection of the Unified Parkinson's Disease Rating Scale (UPDRS-III).

Patients usually completed two conditions in a row. One session comprising two conditions took ~3 h. Patients, who participated in all four stimulation conditions, completed the experiment on two different days. The order of events was kept identical for control subjects, including the 30 minutes breaks between trials to control for tiring effects.

### Electrode positions in standard space

Pre- and post-operative computerized tomography (CT) scans^1^ and pre-operative magnetic resonance imaging (MRI) scans were used to determine the position of the ventralmost contacts of the DBS electrodes used for the unilateral stimulation conditions. Imaging data from one patient had to be excluded from this analysis because the CT scans were too distorted to ensure accurate co-registration.

For each patient the post-operative CT scan was co-registered to the MRI scan in two steps. First, the pre-operative CT scan was aligned with the MRI scan (General Registration BRAINS, implemented in 3DSlicer 4.3.1, www.slicer.org; Fedorov et al., 2012). Since the pre-operative CT was void of electrode artifacts this allowed accurate co-registration. In a second step, the post-operative CT scan was aligned with the co-registered pre-operative CT scan using manual and automatic transformations. For the subsequent normalization and electrode localization we used the MATLAB® Toolbox LEAD DBS (Horn and Kühn, 2015). MRI and CT scans were normalized to MNI (Montreal Neurological Institute) space (SPM New Segment non-linear method, www.fil.ion.ucl.ac.uk/spm/software/spm8, Ashburner and Friston, 2005) and electrode trajectories were reconstructed automatically, visually checked and corrected. To determine whether the positions of the stimulation contact in the ventral conditions were related to individual exploration biases on a group level, a linear regression model (bias ~ β_1_x + β_2_y + β_3_z) was estimated with the demeaned electrode positions as predictors and the z-transformed bias data as predicted variable. The three regression coefficients (β_1_-β_3_) determine a vector along which exploration biases changed most across subjects in MNI space.

### Visuospatial bias

To identify whether viewing behavior was biased, we computed three measures: (1) The median horizontal position of the first fixation in each trial representing the initial bias. (2) The median horizontal position of all fixations after the first one, carrying information about the extent of horizontal deviation. And (3), the exploration time spent on the right hemifield, as assessed by the fraction of spatio-temporal fixation density maps falling onto the right side of the image. The spatio-temporal fixation density maps were 2D maps generated by weighting all fixations after the first one by their duration and then spatially smoothing fixation locations via a 2-dimensional convolution with a Gaussian kernel (std = 0.5° visual angle). This way, both fixation spread and durations were taken into account. Subsequently, we took the median of the trialwise fraction to get robust subject estimates.

### Statistical analyses

To detect disease-related impairments independently from DBS effects, we tested measures obtained in the OFF condition against the average of both runs of control subjects (which were not statistically different). In addition to testing differences between patients in OFF and controls, we tested for within-patients differences between the four DBS conditions. Only when a condition differed from OFF it was further compared with the values of control subjects.

We employed two statistical models of Bayesian analyses: BEST (Kruschke, 2013), as a robust substitute for *t*-tests, and an ANOVA-like extension of its concepts to four conditions (Kruschke, 2011). Model specifications are described in the Supplementary Material. Unlike a *t*-test, BEST uses Bayesian estimation to provide posterior probabilities of group or condition means and mean effect sizes. Results were obtained using R (version 3.0.3, http://www.r-project.org/; R Core Team, 2014) and JAGS (version 3.4.0, http://mcmc-jags.sourceforge.net/; Plummer, 2014) and are presented as group/condition means and their mean differences, both with their respective 95% Highest Density Intervals (HDI) (Kruschke, 2011), and with estimates of mean effect size ((μ_1_ − μ_2_)/√((σ_1_ + σ_2_)/2) when appropriate (Kruschke, 2013). HDIs are defined as the smallest interval of the posterior probability distribution spanning a given fraction (e.g., 95%). This definition is a close analog to the notion of confidence intervals in classical statistics. It provides an intuitive summary statistic for each group/condition mean and mean effect sizes in the sense that all values within the HDI are more likely than any value outside the HDI. We consider differences significant if their 95% HDI exclude zero showing that the probability of the parameter difference being zero is below 5% given the prior of the Bayesian approach. HDIs shown in the figures depict the variability between subjects in individual conditions rather than the condition differences derived from paired comparisons. This resembles classical statistical hypothesis tests, where differences are significant if their 95% confidence interval excludes zero. For ordinal data like UPDRS-III scores, non-parametric tests and Bonferroni-Holm corrected thresholds were applied. All tests were two-tailed. A more detailed description of the Bayesian model can be found in the Supplementary Material.

## Results

We first describe how patients' general motor impairment responded to the altered stimulation settings, and then discuss the effects on oculomotor recordings. Finally, we report about differences in attention with respect to viewing biases.

### Skeletomotor symptoms and DBS

**Figure 2:**
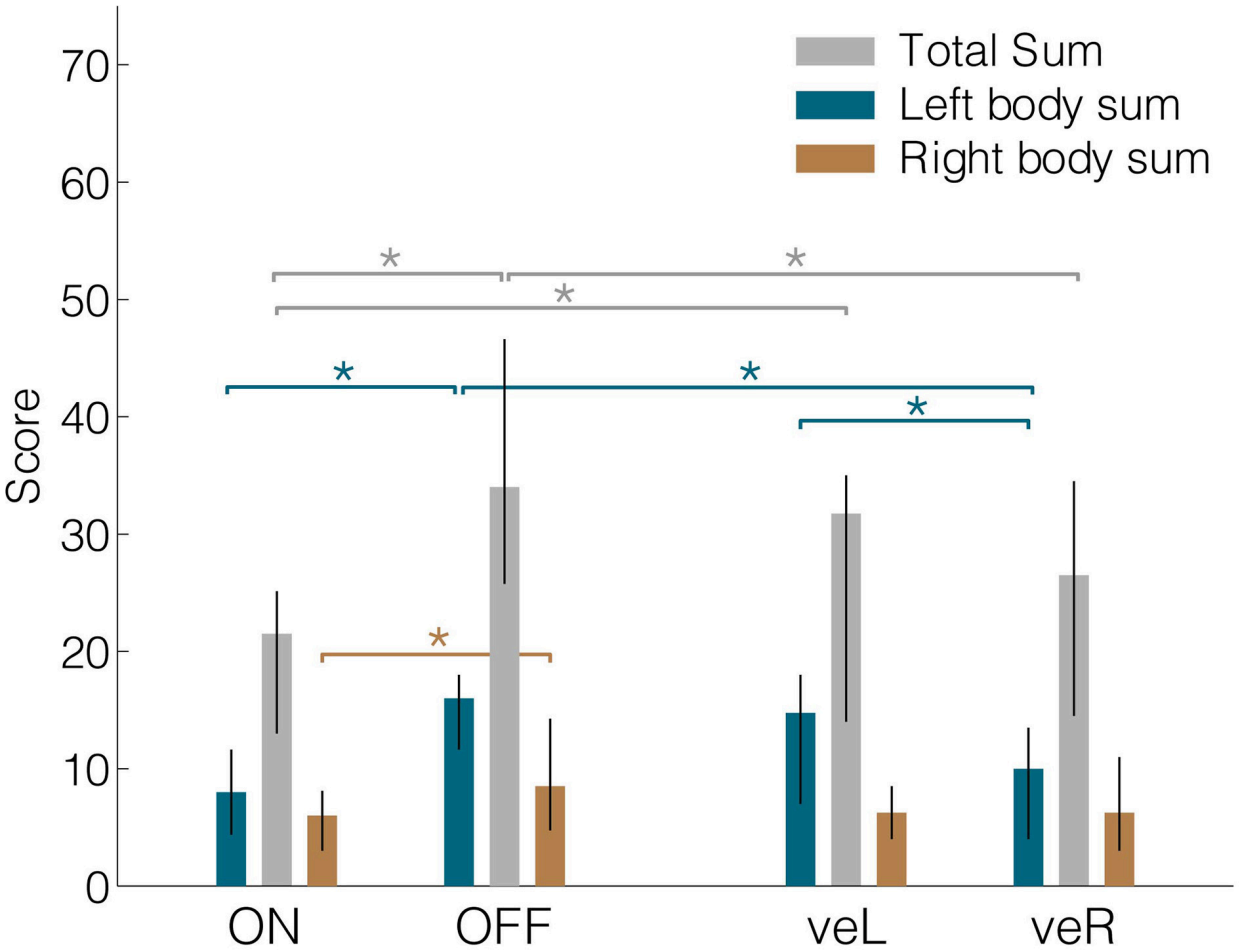
**Median UPDRS-III total sums, left body sums and right body sums for all four conditions**. Lateralized values were derived from items 20–26. ON = DBS On, OFF = DBS OFF, veR = Ventral contact of right electrode active and left electrode off, veL = Ventral contact of left electrode active and right electrode off. Error bars denote the interquartile range. ^*^Denotes significant differences (signed-rank test, *p* < 0.05).

We evaluated differences in patients' UPDRS-III motor scores across the four DBS conditions [Friedman ANOVA, $\chi_{\left(3,39\right)}^{2}$ = 19.07, *p* < 0.001]. As expected, motor symptoms were least pronounced during clinical stimulation (Figure 2, median ON = 21.5; veR = 26.5; veL = 31.8; OFF = 34.0). Pairwise comparisons showed significant differences between ON against OFF (Wilcoxon signed rank test, *p* < 0.001), veR against OFF (*p* < 0.001), and ON against veL (*p* = 0.009). DBS affected the UPDRS-III items of the left body side in a similar manner as it affected the overall sum, i.e., the ordering of scores was identical [Friedman ANOVA, $\chi_{\left(3,39\right)}^{2}$ = 22.47, *p* < 0.001; median ON = 8.0; veR = 10.0; veL = 14.8; OFF = 16.0]. Furthermore, the three pairwise comparisons listed above also yielded a significant improvement of left-sided symptoms (ON against OFF *p* < 0.001; veR against OFF *p* < 0.001; ON against veL *p* = 0.005). Additionally, veR was significantly more effective than veL (*p* = 0.001). For the right body side the ordering of scores was again identical, but the ANOVA did not reach significance [$\chi_{\left(3,39\right)}^{2}$ = 7.62; median ON = 6.0; veR = 6.3; veL = 6.3; OFF = 8.5]. Note that the motor scores for the right body side in all four conditions compare well to the left counterparts in the clinical condition. Less pronounced symptoms on the right side thus imply that the range for therapeutic effects was more limited for this side, leading to a floor effect. In summary, in agreement with previous reports (Hershey et al., 2010) clinical stimulation (ON) led to significant improvements of motor symptoms while unilateral ventral stimulation was only partly effective.

### Eye-movements deficits and the effect of DBS

**Figure 3:**
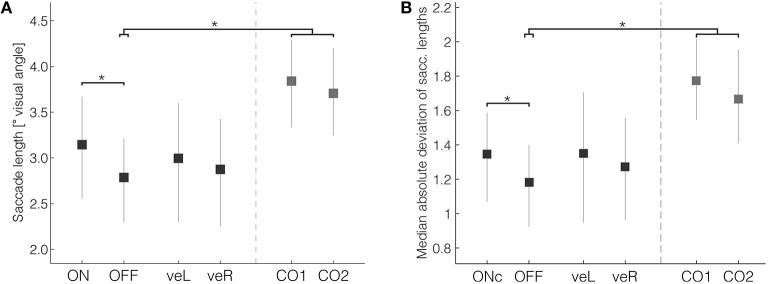
**(A)** Saccade length and **(B)** saccade length variability were significantly reduced in patients when compared with controls. Bilateral DBS in therapeutic settings significantly increased both measures. Error bars are 95% HDIs. ^*^Denotes significant differences (with the 95% HDI of the difference excluding zero).

One of the most consistently affected parameters reported in the literature of eye-movements in Parkinson's disease is reduced saccade length (Vidailhet et al., 1994; Pinkhardt and Kassubek, 2011). In the current experiment, the mean saccade length in patients in the OFF condition (Figure 3A, mean OFF = 2.79°, 95% HDI [2.30, 3.21]) was significantly smaller than the mean of control subjects (mean difference CTRL-OFF = 1.00° [0.36, 1.65], mean effect size = 1.13). The length of saccades did not differ with respect to whether they were directed to the left or right. Clinical DBS resulted in significantly longer saccades in comparison with OFF (mean difference ON-OFF = 0.29° [0.06, 0.52], mean effect size = 0.37). The average saccade length during clinical DBS (mean ON = 3.14° [2.58, 3.68]) was still shorter than that of control subjects, but this difference was not significant (mean difference CTRL-ON = 0.64° [−0.07, 1.35], mean effect size = 0.66). Mean saccade lengths during veR and veL were intermediate between ON and OFF, suggesting only moderate improvement by unilateral ventral stimulation, but those changes were not significant in comparison with ON or OFF. Thus, consistent with previous studies our results demonstrate that clinical DBS partially compensates the reduced saccade length in patients.

As a measure of motor flexibility, which is usually reduced in Parkinson's disease patients (Plotnik et al., 1998; Inzelberg et al., 2001), we evaluated saccade length variation during exploration. Saccade length variability, calculated as the median absolute deviation of the saccade length within each trial, was significantly reduced in patients compared with controls (Figure 3B, mean OFF = 1.18° [0.93, 1.40]); mean difference CTRL-OFF = 0.56° [0.23, 0.90]; mean effect size = 0.94). The variability was, similar as saccade lengths *per se*, significantly improved by ON (mean ON = 1.35° [1.07, 1.58]; mean difference ON-OFF = 0.13° [0.01, 0.25]; mean effect size = 0.36).

To evaluate differences in saccade velocity it is necessary to take into account that saccade peak velocities are closely tied to saccade lengths, with the latter being broadly distributed during free-viewing. A simple comparison of mean peak velocities could therefore be confounded by patients making smaller saccades than controls. In order to control for this, we evaluated subjects' saccade main sequences. The main sequence is the log-log linear relationship between saccades lengths and velocities. To simplify the analysis of slopes and offsets of this linear function, we evaluated the definite integral of the main sequence between 0.1 and 10° visual angle, as the relationship is linear in that range (Bahill et al., 1975). It is of interest to note that saccadic peak velocity, when controlling for their length as assessed via the area under the saccade main sequence, did not differ between patients and controls (mean OFF = 1.03 [0.99, 1.06]); mean difference CTRL-OFF = 0.01 [−0.04, 0.05]; mean effect size = 0.12), or between DBS conditions (mean ON = 1.04 [1.00, 1.07]; mean difference ON-OFF = 0.01 [−0.01, 0.03]; mean effect size = 0.14). In summary, when adjusted for length differences, patients' saccades were not significantly slower than controls.

Furthermore, we also assessed a variety of additional oculomotor parameters, where no difference was found between patients and controls, or between DBS conditions (see Supplementary Table 1). Even though the area viewed (percentage of the complete picture) and the amount of saccades tended to be smaller in patients than in controls, these differences were not significant. Patients' rightward saccades (defined as being directed to the right relative to the previous fixation point irrespective of target hemifield) were not more frequent or longer relative to leftward saccades than in controls. Fixation durations and saccade angle variability were similar across conditions and patient groups. Altogether, these measures indicate that differences in saccade lengths and variability between patients and controls did not cause slower exploration because the number of eye movements and the area covered was not significantly reduced.

In summary, oculomotor parameters under free-viewing conditions were affected in patients in a way that is compatible with previous results obtained in simpler tasks. Patients generally made smaller saccades and showed less saccade length variability than control subjects. Both measures were significantly improved by clinical stimulation such that they became more similar to those of controls.

### Exploration bias in Parkinson's disease

Previous studies have reported subclinical neglect in Parkinson's disease patients, but its impact on patients' daily activities is still unclear. We evaluated if an exploration bias exists during the scanning and exploration of complex natural scenes and how it can be affected by unilateral STN-DBS.

**Figure 4:**
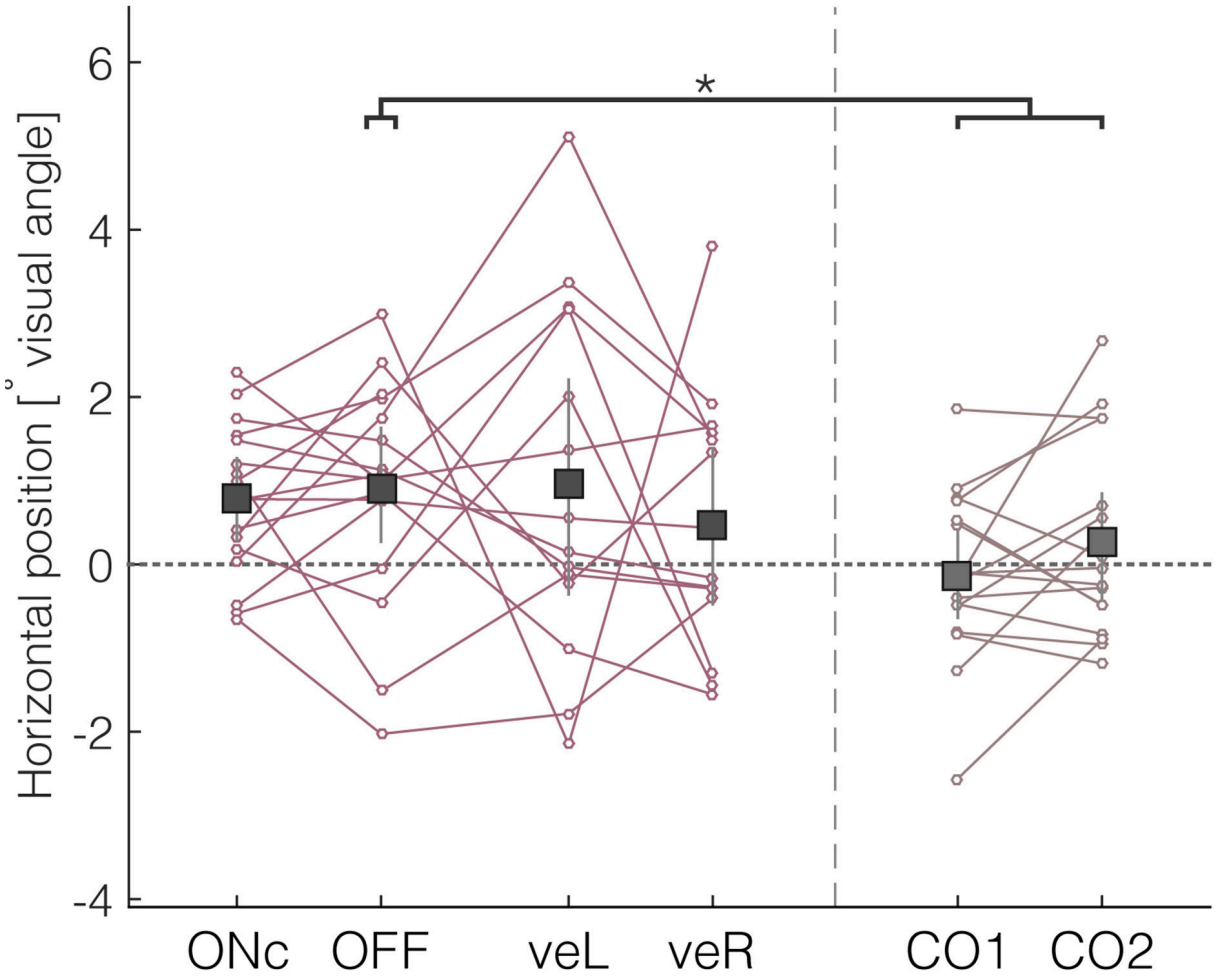
**Rightward bias of patients assessed by the median horizontal position**. Negative values indicate deviations to the left, and positive values deviations to the right. ^*^Denotes significant differences (with the 95% HDI of the difference excluding zero).

Patients' median horizontal fixation position was in OFF significantly shifted to the right as compared with control subjects (Figure 4, mean OFF = 0.89° [0.27, 1.63]; mean difference-OFF-CTRL = 0.88° [0.05°, 1.75°]; mean effect size = 0.78). The four DBS conditions did not differ significantly from each other (mean ON = 0.77° [0.29, 1.27]; mean veL = 0.95° [−0.36, 2.21]; mean veR = 0.48° [−0.48, 1.39]). Patients' percentage of exploration toward the right in OFF was significantly different from 50%, which was not the case for control subjects (mean-CTRL = 52.19% [48.37, 55.53]). The absence of altered fixation durations (Supplementary Table 1) indicates that it is the extent of fixation deviation toward the right hemifield, and not the duration of the fixation that contributed most to patients' rightward bias. There were no significant differences in vertical bias, which was computed similarly to the rightward bias as percentage of summed fixation durations to the upper visual field or as median vertical position (Supplementary Table 1).

During free-viewing of static images, healthy subjects first explore the left side of images (Ossandón et al., 2014). We assessed whether this early bias was also present in our patient group by examining the horizontal position of the first fixation relative to the center. Control subjects tended to direct their first fixation to the left hemifield of the image (mean CTRL = −1.00° [−1.72, −0.23]). In contrast, patients' median horizontal position of the first fixation was centered (mean OFF = 0.14° [−0.52, 0.87]), and hence showed a significant shift to the right in comparison with controls (mean difference OFF-CTRL = 1.15° [0.14, 2.15], mean effect size = 0.82). Again, there were no significant differences between the different DBS conditions.

In summary, we found evidence of a slight general rightward bias in patients, which is consistent with previous reports of attentional bias in patients with Parkinson's disease, and a reduced early leftward bias. In contrast to the reduction of saccadic impairments seen in the previous section, clinical DBS did not change this bias pattern. Contrary to our initial predictions, unilateral stimulation directed at ventral subthalamic regions did neither reduce the bias nor reverse the direction at the group level.

### Individual analysis

**Figure 5:**
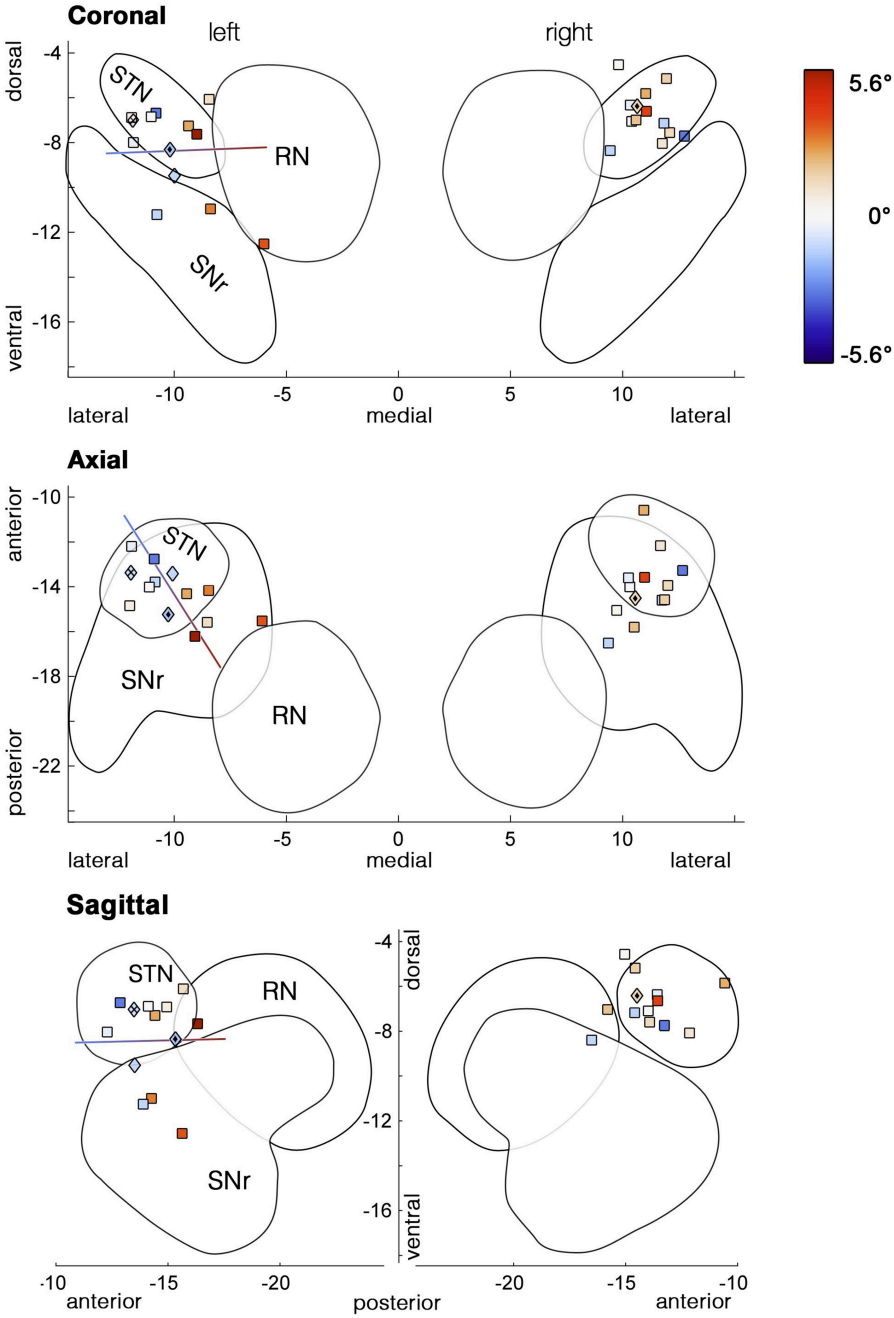
**Stimulation sites of the most ventral contacts used in the unilateral stimulation conditions**. Marker colors code the amount of initial bias (° visual angle) with cool colors representing deviations to the left and warm colors deviations to the right. Stimulation sites, where increased voltage induced torticollis prior to determining the side-effects threshold, are depicted as diamond-shaped markers. The line represents the outcome of the multiple regression model, i.e., the best direction for predicting the initial bias during unilateral left stimulation. Variability of the location along the antero-posterior axis explained most of the bias variance, followed by the medio-lateral direction. Outlines of subcortical structures are based on 3D models of the ATAG Atlas (Keuken et al., 2013).

As the variability between patients appeared to be increased during unilateral stimulation of the ventral subthalamic area (Figure 4), we hypothesized that an effect of unilateral ventral DBS might have been concealed by variability in the electrode position among subjects. We reconstructed the positions of subjects' ventralmost DBS electrode contacts in normalized MNI space. As expected, based on the ATAG Atlas (Keuken et al., 2013) these stimulation sites were concentrated in ventral STN (Figure 5, upper panel), along with more ventral and posteromedial spots in SNr and prerubral area, respectively. We then performed a multiple regression of the measures of bias with the electrode position in three dimensions as predictors. For unilateral left DBS, the bias of the first movement showed a significant dependence on the position of the electrode both for the absolute condition bias [Figure 5, *F*_(3, 10)_ = 4.2, *p* = 0.037, *r*^2^ = 0.56] and for the relative difference between veL and OFF [*F*_(3, 10)_ = 4.0, *p* = 0.041, *r*^2^ = 0.54]. Considering the global bias measured by median horizontal fixation positions, the models for the absolute bias in veL and the condition difference veL-OFF were only close to significant [*F*_(3, 10)_ = 3.3, *p* = 0.067, *r*^2^ = 0.50, relative condition difference veL-OFF: *F*_(3, 10)_ = 2.7, *p* = 0.100, *r*^2^ = 0.45]. Figure 5 shows the resulting regression line, indicating that the observed initial bias was dependent on where along this axis the stimulating contact was located. It mainly extends along the posterior-anterior direction, followed by the medial-lateral direction (upper panel).

While in both unilateral conditions more than half of all patients spent ~50% of their exploration time on each hemifield, some patients were strongly biased toward one side (see Table 2 or Figure 5). One of those patients (#13) showed stimulation-induced torticollis upon identifying the voltage threshold for side effects. His head turned toward the ipsilateral side of stimulation, i.e., to the right upon ventral right stimulation. In a second patient we observed a head turn to the left upon increasing the voltage for veL (#11). Voltage was lowered until the muscle contractions fully receded, yet their fixations were still shifted toward the turning direction (Table 2). Interestingly, both patients received stimulation at similar sites—at the posterior border of the STN (Figure 5, 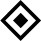 markers). Remarkably, another patient's head (#1, 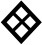) involuntarily turned to the contralateral side upon determining the voltage threshold for unilateral left stimulation. Even though his head was turning to the right, his gaze was deviating toward the left. During the subsequent experiment he explored predominantly his left visual field, but surprisingly his rightward saccades were notably longer than his leftward saccades in comparison with most other patients (Table 2, ΔSac). The length of saccades seemed to be biased toward the direction of the neck torsion and dissociated from the leftward deviation of the gaze and the left-directed exploration bias. A fourth patient's head (#4, ◇) turned slightly to the right when increasing the voltage prior to veL, but this patient showed no general viewing bias (51.9%).

## Discussion

Bilateral clinical and unilateral ventral right DBS (applied contralateral to the initially most affected body side) alleviated patients' left-dominant skeletomotor symptoms. In contrast, patients' saccades were not lateralized (e.g., more frequent or longer when directed to the right), and were significantly improved only by bilateral stimulation. The extent of the improvements (~40%) was similar to the change in motor symptoms measured with the UPDRS-III scale (~37%), indicating similarities between the responsiveness of the skeletomotor and oculomotor systems. However, patients' exploration bias displayed during OFF was not counteracted by clinical stimulation. Only unilateral left stimulation seemed to modify biased viewing behavior at the beginning of exploration; this effect was not apparent at the group level since it was dependent on the precise electrode location within the ventral subthalamic area.

### Limitations

This paper provides a set of observations on the basis of explorative data analysis. One limitation of our study is that neither patients nor experimenters were blinded to the experimental conditions because the threshold for side effects in the unilateral stimulation condition had to be determined. However, patients were not aware of the hypotheses of our study.

Visuospatial attention deficits might be more pronounced after medication withdrawal. However, we had to renounce initial plans to conduct the study after withdrawal of antiparkinsonian medication because discomfort resulting from severe Parkinson's disease symptoms caused a large drop-out rate after the first recording session. Furthermore, three patients showed no deterioration in motor symptoms when stimulation was off, which might be related to fluctuations at different day times or fatigue after completing the ON recording, and might have reduced our statistical power.

Finally, the order of conditions was not fully balanced given that the first recording in 8 of the 17 patients (and 6 of the 14 recorded in four conditions) was performed ON DBS to reduce our patients' expenditure of time. Task novelty might have affected behavior, however, we would not expect any consistent changes in free-viewing related to learning over time.

### Oculomotor impairments in Parkinson's disease

This positive effect of clinical STN-DBS on saccade length is in line with previous reports of improvement by DBS in saccade length during volitional memory-guided tasks (Rivaud-Péchoux et al., 2000; Fawcett et al., 2010), anti-saccade tasks (Briand et al., 1999; Fawcett et al., 2010; Yugeta et al., 2010), smooth pursuit (Nilsson et al., 2013), and visually triggered saccade tasks (Sauleau et al., 2008; Yugeta et al., 2010). As early neurophysiological studies in monkeys showed no involvement of the basal ganglia in spontaneous exploration (Hikosaka and Wurtz, 1983), it was not certain that effects seen in those simple tasks would generalize to free-viewing behavior. Only recent studies in humans (Sieger et al., 2013) are more in line with our findings, showing that the basal ganglia are active during free viewing, and that exploratory visuomotor behavior thus can be affected in Parkinson's disease as a consequence of basal ganglia dysfunction.

Our results extend a previous report about free-viewing behavior and the effects of DBS in patients with Parkinson's disease (Schmalbach et al., 2014). In agreement with their results, we did not find significant changes in saccade lengths between bilateral and unilateral stimulation conditions. However, as we included a healthy control group and patients in a baseline condition without DBS, we were able to show that saccade length during free-viewing is reduced in the parkinsonian state and improved by clinical stimulation. Furthermore, patients' within-trial saccade length variability was reduced in comparison with controls. This variability was increased by bilateral DBS to a level more similar to that of controls. However, the impact of these oculomotor alterations on behavior is not clear since the total explored area was not reduced.

### Exploration bias during free-viewing behavior

In agreement with previous research showing attentional biases in patients with left-dominant symptoms in other tasks (Villardita et al., 1983; Starkstein et al., 1987; Ebersbach et al., 1996; Lee et al., 2001; Laudate et al., 2013), our patients also showed a slight rightward bias during free-viewing. The difference to control subjects was most pronounced during the first eye movements, which are usually biased to the left in healthy right-handers (Ossandón et al., 2014). This attentional bias was neither explained by a directional bias in saccade lengths (i.e., rightward saccades were not significantly longer or more abundant than leftward saccades) nor by differences in fixation durations between hemifields. The exploration bias was not compensated by clinical stimulation even though stimulation decreased markedly the discrepancy between left and right motor symptoms. In the study by Schmalbach et al. (2014), Parkinson's disease patients did not show biased free-viewing behavior during clinical stimulation. This discrepancy may be explained by the small size of the bias, which became evident only by comparison with a control group. Moreover, Schmalbach et al. (2014) included patients with both left- and right-dominant symptoms whereas we evaluated only patients with left hemibody onset of symptoms, who are more likely to show attentional bias (Villardita et al., 1983; Starkstein et al., 1987; Ebersbach et al., 1996; Lee et al., 2001; Laudate et al., 2013). As mentioned above, the bias was not improved by clinical bilateral stimulation like some of the oculomotor parameters, potentially suggesting differences in the role of the basal ganglia in skeletomotor, oculomotor and attentional functions. This discrepancy may result from differences in topographic organization of these functions within the STN, which remains to be elucidated (Alkemade et al., 2015).

Contrary to our expectations, unilateral stimulation in the ventral subthalamic area caused no consistent shifts of viewing biases but higher variability. A rightward bias after left STN-DBS as reported by Schmalbach et al. (2014) would have been in agreement with the assumption of a disinhibitory effect of DBS and the known circuitry of the basal ganglia ocular movement control. In this model, inhibition of the STN results in decreased excitatory input to the SNr and consequently in a reduced inhibitory tone to the ipsilateral superior colliculus—biasing viewing behavior to the contralateral right visual field. Although we did not find an effect of unilateral stimulation at the group level, the multiple regression analysis revealed a dependency between bias and the position of the activated electrode contact within the left STN. Those patients who were stimulated in the posterior part of the STN showed a stronger bias toward the right. To understand this relationship it is necessary to look into the fine-grained topographic organization of the basal ganglia circuit, which is still not completely clear, especially in humans. Previous studies in non-human animals showed that the efferents from the STN to the SNr are similarly arranged, especially in the latero/medial axis (Smith et al., 1990; Joel and Weiner, 1994; Parent and Hazrati, 1995). Additionally, in monkeys and cats, projections from the SNr to the superior colliculus include abundant uncrossed and broadly distributed projections, as well as less numerous crossed projections to the contralateral SC departing from the anterolateral SNr (Beckstead et al., 1981; Jiang et al., 2003). Assuming a similar organization of the human STN/SNr complex, more anterior STN stimulation (i.e., disruption of pathological hypersynchronization) should result in reduced activity of the corresponding anterolateral SNr, with an impact on both crossed and uncrossed projections. In contrast, more posterior stimulation of the STN should predominantly result in reduced activation of only the uncrossed population. This would cause, in the case of ventral left posterior stimulation, a bias to the right due to exclusive disinhibition of the ipsilateral SC, whereas more anterior stimulation would result in more balanced disinhibition of both SC and consequently no bias. Yet it is unclear why this occurs only for left stimulation and why it affected predominantly the first fixation of each trial.

An important question is whether the exploration biases reported here represent an inability to detect and respond to stimuli and whether this happens to an extent that would affect them in everyday activities. The average bias observed at the group level was rather small, and thus has probably only limited impact on daily life. Nonetheless it could impact patient's ability to drive (e.g., Uc et al., 2006; Classen et al., 2014) and increase the incidence rate of bumping into objects (Davidsdottir et al., 2005). Individuals might experience more pronounced visuospatial deficits, which is why individual assessments of driving capabilities would be advisable (Buhmann et al., 2014). Clinical DBS provides no simple remedy for such impairments, yet trying to select different electrode contacts for stimulation might restore balanced attention and recover normal exploration behavior. On the other hand, considering the association between posterior sites and bias presented here, we would recommend to routinely evaluate whether an attentional bias may have been induced by DBS, especially in rare cases of unilateral stimulation or when the posterior subthalamic area is targeted as has been suggested for the treatment of tremor suppression (Power et al., 2001; Plaha et al., 2006; Xie et al., 2012).

In summary, patients with Parkinson's disease made shorter saccades with a reduced length variability, and were slightly biased toward the right in comparison with controls. Saccade length was significantly improved by clinical stimulation, whereas the effects of unilateral stimulation of the ventral subthalamic area on exploration were dependent on the individual stimulation site and not seen at the group level. The findings presented provide new evidence for the involvement of the basal ganglia in self-directed visual exploration and will hopefully guide further research on the treatment of patients with visuospatial attention deficits or cervical dystonia.

## Author contributions

CKEM, JPO, J Keyser, AKE and PK designed the study. WH, MW, J Köppen, AG and CKEM performed deep brain stimulation surgeries. CKEM, AG, CB, MW, CG contributed to patient recruitment. PF, J Keyser, CKEM, AG and JPO collected the data. PF, JPO, J Keyser, NW, CKEM and PK performed or supervised data analysis. All authors contributed in writing and revising the paper.

## Funding

This study was supported by funding from the EU (FP7- ICT-270212, ERC-2010-AdG-269716, AKE, PK) and the DFG (SFB936/A3/B6/C1/C8, AKE, PK, CG, AG, CKEM).

### Conflict of interest statement

AG, WH, CB, and CM have occasionally received travel reimbursement by Medtronic Inc. The other authors declare that the research was conducted in the absence of any commercial or financial relationships that could be construed as a potential conflict of interest.

## Footnotes

^1^CT scans obtained with a Somatom Sensation 16 (Siemens, Germany).
