# Supplementary material for: STN-DBS Reduces Saccadic Hypometria but Not Visuospatial Bias in Parkinson's Disease Patients
Source: Front Behav Neurosci. 2016 May 3;10:85. doi: 10.3389/fnbeh.2016.00085 (PMC4853960; doi:10.3389/fnbeh.2016.00085)
Supplement: Supplementary file 1 [file Table1.doc]

Supplementary Material

**Amplification of attentional bias in Parkinson’s disease by unilateral STN-DBS during free-viewing**

**Petra Fischer^1,2,3*^, José P. Ossandón^1*^, Johannes Keyser^1^, Alessandro Gulberti^4^, Niklas Wilming^1,4^,Wolfgang Hamel^5^, Johannes Köppen^5^, Carsten Buhmann^6^, Manfred Westphal^5^, Christian Gerloff^6^, Christian K.E. Moll^4^, Andreas K. Engel^4^, Peter König^1,4^**

*** Correspondence:** Corresponding Authors: [pefischer@uos.de](mailto:pefischer@uos.de), [c.moll@uke.de](mailto:c.moll@uke.de)

# Supplementary Figures and Tables

|  | ON | OFF | veL | veR | CTRL 1 | CTRL 2 |
| --- | --- | --- | --- | --- | --- | --- |
| Area viewed (%) | 17.3 ±3 | 17.2 ±4 | 16.9 ±4 | 16.2 ±5 | 19.7 ±3 | 18.9 ±4 |
| Fixation Duration | 265 ±35 | 255 ±32 | 258 ±41 | 265 ±48 | 261 ±23 | 260 ±29 |
| Fixation Duration Ri-Le | 5.2 ±13 | 3.3 ±19 | 10.5 ±21 | 6.0 ±25 | -0.1 ±14 | 2.2 ±9 |
| SMS Peak Velocity Integral | 1.04 ±0.1 | 1.03 ±0.1 | 1.03 ±0.1 | 1.03 ±0.1 | 1.04 ±0.1 | 1.03 ±0.1 |
| Number of Saccades | 682 ±105 | 691 ±132 | 690 ±129 | 647 ±154 | 739 ±98 | 716 ±116 |
| % Rightward Saccades | 49.7 ±3 | 50.6 ±4 | 50.5 ±5 | 50.4 ±3 | 49.4 ±4 | 49.8 ±3 |
| Saccade Length Ri-Le | 0.06 ±0.4 | 0.02 ±0.4 | 0.20 ±0.6 | -0.08 ±0.4 | 0.17 ±0.6 | 0.15 ±0.4 |
| Saccade Angle Variability | 1.79 ±0.1 | 1.82 ±0.1 | 1.80 ±0.1 | 1.80 ±0.1 | 1.82 ±0.1 | 1.79 ±0.1 |
| Median vertical position | 0.56 ±0.8 | 0.60 ±1.4 | 0.86 ±1.5 | 0.68 ±1.3 | 0.59 ±0.9 | 0.87 ±0.8 |
| Upward bias | 59.5 ±11 | 58.5 ±15 | 57.9 ±14 | 60.3 ±17 | 55.7 ±7 | 56.3 ±9 |
|  |  |  |  |  |  |  |

**Supplementary Table 1:** Means and standard deviations of eye-movement measures that did not differ between conditions and subject groups (SMS = Saccade Main Sequence, Fixation Duration Ri-Le = Difference between fixations durations on the right hemifield and durations on the left hemifield, Saccade Length Ri-Le = Difference between lengths of rightward saccades and leftward saccades).

**Supplementary Figure 1:** Exemplary photographs taken after the recordings to monitor unrestricted eye motility. Participants shifted their gaze into nine different directions without moving their head.

# Bayesian model specification

Several reasons led to the choice of robust Bayesian statistics for drawing inference from our data: First, they allow to present the data in a comprehensive manner in terms of parameters' joint posterior distributions. These can quantitatively inform future experiments as priors, which is especially valuable for patient studies, where data acquisition often comes at high costs. Secondly, the models are robust with respect to moderately non-normal data, such as those presented here. Furthermore, outliers can be accounted for by incorporating adaptable Student's *t*-distributions as likelihood functions. Depending on the input data, the heavy tails of the *t*-distribution are increased in case of outliers, or attenuated to approximate a normal distribution if there are none. Thirdly, separate variance model parameters for each condition remove the assumption of equal variances across conditions. Finally, the BANOVA model (Kruschke, 2011) provides an in-built solution for the multiple comparison problem by virtue of an overarching distribution across the condition effects: Similar mean parameters among conditions lead to smaller estimated variability between them, and thus cause „parameter shrinkage“, i.e. different condition estimates get drawn towards the overarching distributions' mean (Gelman, Hill, & Yajima, 2012; Kruschke, 2013). This is important as our experimental design required six pairwise comparisons to test for differences between all combinations of the four DBS conditions.

Posterior distributions of model parameters were estimated to fit our data within the Bayesian framework using R (version 3.0.3, <http://www.r-project.org/>, R Core Team (2014)). For one- and two-sample comparisons of means, we used the BEST model (Kruschke 2013), and for comparisons across conditions an ANOVA-like extension of its concepts to four conditions (Kruschke, 2011). In the model specification, data result from Student's *t* distributions (y[i] ~ dt(*µ*[i] , 1/σ^2 , nu)) with a mean parameter determined by *µ*[c,s] ~ m0 + m1[c] + m2[s], where m0 denotes the overall mean, m1 the condition means (i.e. stimulation effects), and m2 the individual subject means (i.e. a "nuisance" factor modeling additive differences attributable to each subject). Model parameters were fit to the z-transformed data, and then back-transformed. The conditions' and subjects' effects on the mean were restricted to sum to zero, respectively. Priors for estimated effects m0, m1's and m2's were set to vague Gaussian priors around zero. Variances were estimated for each condition with a uniform distribution σ ~ U(0,10) as prior. The prior for the “normality parameter”, i.e. the *t*-distribution’s degree of freedom, was an exponential distribution (nu ~ exp(1/29)+1) to incorporate approximately even prior credibility of nearly-normal and heavy-tailed data (Kruschke, 2013). Posterior distributions were approximated using Markov Chain Monte Carlo (MCMC) sampling from four parallel chains. Models were specified for JAGS (version 3.4.0, <http://mcmc-jags.sourceforge.net/>; Plummer, 2014), interfaced to R via the rjags package, version 3-13 (Plummer, 2014) and executed by runjags version 1.2.0-7. Adaptation steps were 1000 for all models, followed by a burn-in period of 20000 steps and 150 000 final saved samples. No thinning was applied. Convergence was assessed graphically and by ensuring (multivariate) potential scale reduction factors (psrf) below 1.05 for all parameters (Brooks & Gelman, 1998). After passing the criterion of a psrf below 1.05, the four chains were combined.

**References Supplementary Material**

Brooks, Stephen P, Gelman A “General Methods for Monitoring Convergence of Iterative Simulations.” Journal of Computational and Graphical Statistics 7, no. 4 (December 1998): 434–55. doi:10.1080/10618600.1998.10474787

Gelman A, Hill J, Yajima M (2012). Why we (usually) don’t have to worry about multiple comparisons. Journal of Research on Educational Effectiveness, 5, 189–211. doi:10.1080/19345747.2011.618213

Kruschke J (2011). Doing Bayesian data analysis: A tutorial introduction with R. Burlington, MA: Academic Press.

Kruschke J (2013). Bayesian Estimation Supersedes the t Test. Journal of Experimental Psychology: General, 142(2), 573–603. doi:10.1037/a0029146

R Core Team (2014). R: A language and environment for statistical computing. R Foundation for Statistical Computing, Vienna, Austria. URL <http://www.R-project.org/>

Plummer M (2014). rjags: Bayesian graphical models using MCMC. R package version 3-13. http://CRAN.R-project.org/package=rjags
