# Supplementary figures and images for: STN-DBS Reduces Saccadic Hypometria but Not Visuospatial Bias in Parkinson's Disease Patients
Source: Front Behav Neurosci. 2016 May 3;10:85. doi: 10.3389/fnbeh.2016.00085 (PMC4853960; doi:10.3389/fnbeh.2016.00085)

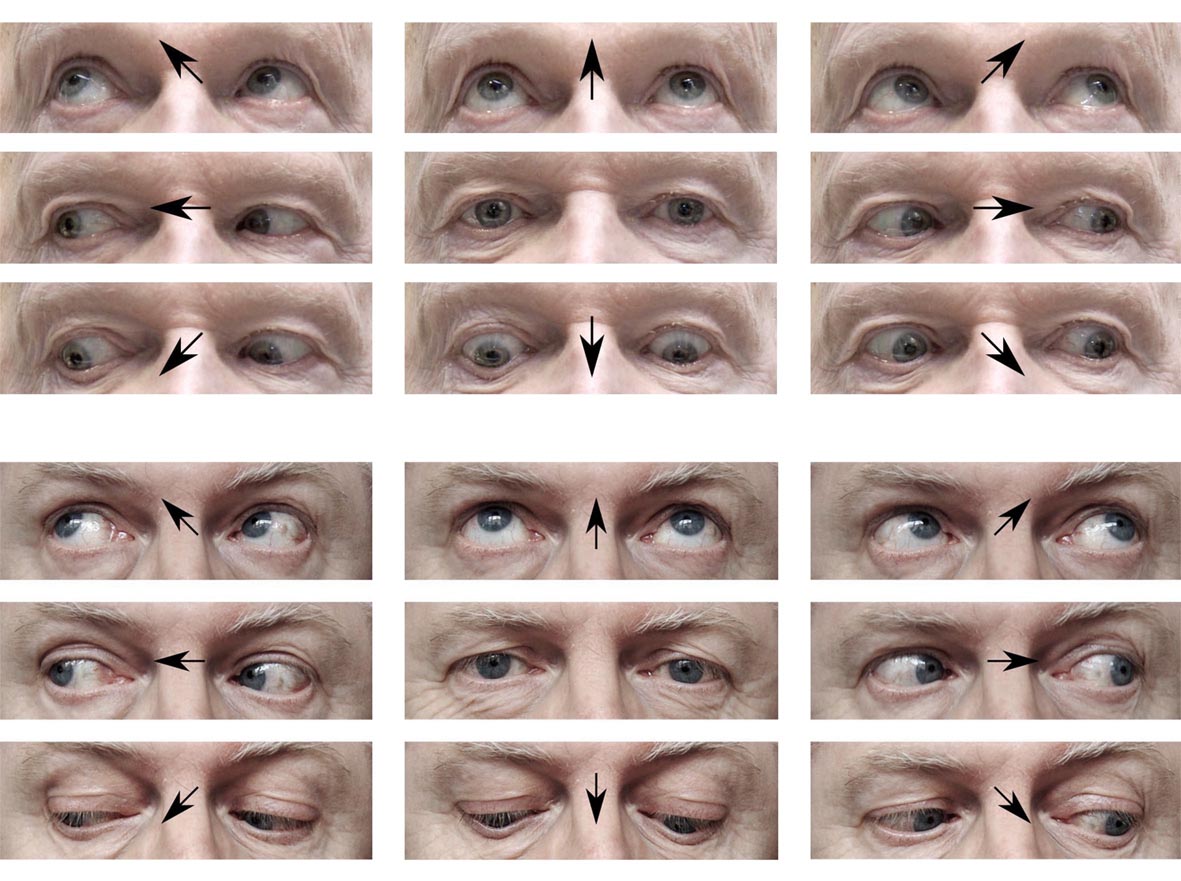

Supplement: Supplementary file 2 [file Image1.JPEG]
